# Fermented strawberry pomace enhances carcass characteristics and meat quality by regulating plasma biochemical indices and antioxidant capacity in aged laying hens

**DOI:** 10.3389/fnut.2025.1610660

**Published:** 2025-06-19

**Authors:** Wei Lan, Ting Chen, Binghua Qin, Md. Abul Kalam Azad, Zhihua Li, Yadong Cui, Xiangfeng Kong

**Affiliations:** ^1^School of Biology and Food Engineering, Fuyang Normal University, Fuyang, Anhui, China; ^2^Anhui Engineering Research Center for Functional Fruit Drink and Ecological Fermentation, Fuyang, Anhui, China; ^3^College of Food and Nutrition, Anhui Agricultural University, Hefei, Anhui, China; ^4^Hunan Provincial Key Laboratory of Animal Nutritional Physiology and Metabolic Process, Institute of Subtropical Agriculture, Chinese Academy of Sciences, Changsha, Hunan, China; ^5^College of Advanced Agricultural Sciences, University of Chinese Academy of Sciences, Beijing, China; ^6^Anhui Jinmu Feed Co., Ltd., Fuyang, Anhui, China

**Keywords:** antioxidant capacity, carcass characteristics, fermented strawberry pomace, laying hens, meat quality, plasma biochemical indices

## Abstract

**Introduction:**

Strawberry pomace, a byproduct of strawberry processing, is rich in vitamins, polyphenols, and other bioactive compounds that may confer beneficial effects on laying hens. This study systematically evaluated how dietary supplementation with fermented strawberry pomace (FSP) modulates carcass characteristics, meat quality, and plasma parameters (lipid profiles and antioxidant capacity) in aged laying hens.

**Methods:**

The study comprised 320 healthy 345-day-old laying hens, which were randomly assigned to four dietary groups receiving 0, 0.25, 0.5%, or 1.0% FSP for 9 weeks.

**Results:**

The 0.25% FSP group showed increased dressing percentage (*p* < 0.05) compared to other groups. FSP supplementation at 0.25–1% reduced breast muscle pH at 48 h post-slaughter, as well as 48 h drip loss. An elevation in leg muscle a* values (*p* < 0.05) was observed in both 0.25 and 0.5% FSP groups compared with the control group, while L* value was higher (*p* < 0.05) in the 0.25% FSP group compared to the 0.5% FSP group. The 0.5% FSP group showed higher leg muscle pH at 45 min (*p* < 0.05) than the control and 0.25% FSP groups. Plasma total antioxidant capacity (T-AOC) was enhanced (*p* < 0.05) in the 1% FSP group compared to the control and 0.25% FSP groups. The 1% FSP group had a higher plasma GLU level (*p* < 0.05) compared with other groups. The plasma FFA level was decreased (*p* < 0.05) in all treatment groups than the control group. All FSP groups exhibited a decreasing trend in plasma TG level (*p* = 0.057). In breast muscle, MDA level was lower (*p* < 0.05) in the 0.25% FSP group compared to the 1% FSP group. In addition, the expression of *SOD1* in breast muscle was up-regulated (*p* < 0.05) in the 0.5% FSP group compared to the 0.25% FSP group.

**Discussion:**

Collectively, dietary FSP supplementation increased carcass characteristics and meat quality by enhancing antioxidant capacity and plasma lipid metabolism in aged laying hens. The low-dose FSP level showed the most favorable outcomes.

## 1 Introduction

In many Asian countries, poultry meat is a source of animal protein, prized for its distinctive flavor and aroma, which makes it a popular choice for preparing aromatic and savory broths (1). However, as laying hens age, their welfare deteriorates rapidly (e.g., lameness, contact dermatitis, and thermal discomfort), and muscle quality declines, leading to reduced consumer acceptance and economic viability in poultry production (2). This decline may be linked to a weakened antioxidant capacity in aged chickens (3). Therefore, improving the meat quality of aged hens after culling could enhance its market competitiveness.

Feed cost has long been a major concern in local poultry production. As poultry production costs continue to rise due to fluctuating prices of essential feedstuffs, such as soybean and corn, it is crucial to develop alternative feed sources. Utilizing agricultural by-products may help to achieve more sustainable resource utilization. Globally, more than 0.5 billion tons of by-products are produced annually from fruit processing, containing valuable nutrients that could serve as potential natural feed resources (4). As a by-product of strawberry juice processing, strawberry pomace contains high levels of dietary fiber, vitamins, minerals, polyphenols, anthocyanins, and ellagitannins (5). It also maintains stable antioxidant capacity (6). Therefore, incorporating strawberry pomace as a feed supplement for livestock and poultry not only helps utilize agricultural by-products but also provides inexpensive nutrients, lowering feed costs, and reducing overall production expenses.

However, strawberry pomace contains high-water content, which makes it prone to mold contamination and difficult to store, potentially leading to environmental pollution (5). Solid-state fermentation helps agricultural by-products last longer and become more nutritious (7). Recent studies have shown that microbial fermentation of agricultural by-products produces organic acids, heteropolysaccharides (e.g., xanthan and chitosan), bioethanol, enzymes, aroma compounds, antioxidants, and other nutrients (8). Consequently, microbial fermentation increases metabolite, enzyme, and probiotic bacteria content, reduces crude fiber, and enhances crude protein in the feed (9). Several studies have reported that fermented feed improves broiler production, nutrient utilization, and meat quality (10, 11). Additionally, a previous research has indicated that dietary supplementing fermented blueberry pomace improves production performance, egg composition, follicle development, and ovarian function in aged laying hens (12). Based on these potential benefits of fermented fruit pomace, we hypothesized that adding fermented strawberry pomace (FSP) to laying hen diets could enhance laying performance, slaughter outcomes, and meat quality by increasing antioxidant capacity. Therefore, the present study aimed to evaluate the effects of dietary FSP supplementation on carcass characteristics, meat quality, plasma biochemical parameters, and the antioxidant capacity in plasma and breast muscle in aged laying hens. Our findings provide a foundation for future studies exploring novel feeding resources.

## 2 Materials and methods

### 2.1 Preparation of FSP

Strawberry pomace was purchased from the Fuyang Fruit Wine Engineering Technology Center (Fuyang, China). The FSP was obtained by fermenting strawberry pomace with a mixture of starters. The conditions and processes of fermentation were in accordance with our previous study (12). The nutritional composition of the strawberry pomace and FSP, including conventional nutrient, amino acids, and fatty acids, is listed in Table 1.

**Table 1:** The nutrient content of the strawberry pomace before and after fermentation.

Component	Before fermentation (%)	After fermentation (%)
Conventional nutrient		
Calcium	0.02	3.16
Crude ash	0.40	11.20
Crude fiber	0.80	21.30
Crude protein	1.10	9.52
Ether extract	0.60	5.30
Gross energy (MJ/kg)	**–**	16.45
Magnesium	0.02	**–**
Amino acid		
Alanine	0.05	0.46
Arginine	**–**	0.36
Aspartic acid	0.08	0.82
Cysteine	**–**	0.14
Glutamic acid	0.08	1.25
Glycine	0.04	0.45
Histidine	–	0.17
Isoleucine	0.04	0.33
Leucine	0.06	0.61
Lysine	0.05	0.34
Methionine	0.02	0.13
Phenylalanine	0.04	0.45
Proline	**–**	0.43
Serine	0.04	0.39
Threonine	0.04	0.34
Tryptophan	**–**	0.04
Tyrosine	**–**	0.21
Valine	0.05	0.46
Total AA	0.71	7.25
Fatty acid		
Palmitic acid (C16:0)	0.02	13.88
Stearic acid (C18:0)	**–**	3.09
Oleic acid (C18:1n-9)	0.02	29.67
9,12-Linoleic acid (C18:2n-6)	0.01	42.53
Linolenic acid (C18:3n-3)	**–**	8.68
Arachidic acid (C20:0)	**–**	1.15
cis-11-Eicosenoic acid (C20:1n-9)	**–**	0.27
Behenic acid (C22:0)	**–**	0.38
Lignoceric acid (C24:0)	**–**	0.36

### 2.2 Animals, experimental design, and diets

A total of 320 Yukou Jingfen No. 8 laying hens (345-day-old) were randomly allocated into four experimental groups. Each group consisted of eight replicates, with 10 hens per replicate. All birds were fed a basal diet (Table 2) for a 7-day adaptation period before the formal feeding trial. The hens in the control group were fed a basal diet, whereas the hens from three experimental groups received the same basal diet added with 0.25, 0.5%, or 1% FSP, respectively. The trial lasted for nine weeks. The hens were kept in wire cages (five birds per cage) with *ad libitum* access to water and feed. The experimental room was well-ventilated, with a humidity range of 45–60%, a controlled temperature of 18–24°C, and an 8/16 h light/dark cycle.

**Table 2:** Composition and nutrient levels of the basal diet (fed basis, %).

Ingredients	Content (%)	Nutrients^2^	Level (%)
Corn	64.20	Metabolizable energy (MJ/kg)	11.38
Soybean meal	21.60	Crude protein	15.56
Limestone	8.00	Ether extract	5.10
Soybean oil	1.20	Crude ash	11.00
Premix^1^	5.00	Calcium	3.52
Total	100.00	Total phosphorus	0.42
		Methionine	0.33
		Lysine	0.95

1 Premix supplied per kilogram of diet: vitamin A, 10,000 IU; vitamin D_3_, 3,000 IU; vitamin E, 20 IU; vitamin K_3_, 1.75 mg; vitamin B_1_, 2 mg; vitamin B_2_, 6 mg; vitamin B_6_, 3 mg; vitamin B_12_, 0.02 mg; nicotinamide, 40 mg; pantothenic acid, 8.5 mg; folic acid, 1 mg; biotin, 0.24 mg; Fe, 75 mg; Cu, 8 mg; Zn, 65 mg; Mn, 100 mg; I, 0.8 mg; Se, 0.3 mg; NaCl, 3 g; Choline chloride, 450 mg; Lys, 1.3 g; Met, 0.95 g; Ca, 5 g.

2 Metabolizable energy is a calculated value, and other nutrient levels are measured values.

### 2.3 Sample collection

When the feeding trial ended, after a 12-h fasting period, eight hens per group were selected based on average body weight. Blood (10 mL) was sampled through wing vein puncture into heparin sodium anticoagulant tubes, and then subjected to centrifugation at 3000 × g for 10 min to separate the plasma, which was subsequently stored at −20°C until analysis. The hens were immediately slaughtered by mechanical cervical dislocation and subsequently dissected to determine carcass characteristics and organ index. Breast and whole leg muscles were separated, weighed, and kept at 4°C for meat quality assessment. Breast muscle tissues (mid-region of *Pectoralis major*) from eight hens per group were quickly frozen in liquid nitrogen and then stored at −80°C for further gene expression analysis related to antioxidant capacity. Breast muscle samples were fixed with 4% paraformaldehyde for morphological observation.

### 2.4 Determination of carcass characteristics and organ index

Carcass characteristics and organ index were determined according to the “Terms and Statistics of Poultry Production Performance” (NY/T823-2020). Prior to slaughter performance analyses, live body weight was recorded following a 12-h fasting period to standardize physiological conditions. Indicators included live body weight, slaughter weight, eviscerated weight, and dressing percentage, as well as the percentages of eviscerated yield, leg muscle, and breast muscle. The organ index was calculated by dividing the organ weight (g) by live body weight (kg) (13, 14).

### 2.5 Determination of meat quality

Meat color (lightness [L*], redness [a*], and yellowness [b*]) of the right breast and leg muscles was measured 45 min post-mortem using a MiniScan XE Plus colorimeter (Hunter Associates Laboratory, Reston, VA, United States). The pH was analyzed with a hand-held pH meter (pH-Star, Matthäus, Pöttmes, Germany) at 45 min and 48 h post-mortem by inserting the electrode parallel to the muscle fibers. Drip loss was measured using previously described methods by Honikel (15). Briefly, a 10 g muscle sample was packaged in a plastic bag, maintained at 4°C, and weighed following 24 and 48 h to calculate drip loss using the following formula:


$$\text{Drip loss}\;(\%)=(W_{1}–W_{2})/W_{1}\times 100.$$

Where W_1_ is the initial sample weight and W_2_ is the final sample weight.

Cooking loss was determined by removing fat and other tissues from approximately 15 g of muscle, and cooked to 80°C until the center temperature of meat reached at 75°C. Cooking loss percentage was calculated as:


$$\begin{array}{l} \text{Cooking loss}\;(\%)\\ =[(\begin{array}{l} \text{fresh weight before cooking}\\ –\text{final weight after cooking} \end{array})/\text{fresh weight before cooking}]\times 100. \end{array}$$

Shear force was assessed using a texture analyzer (TMS-Pro, Food Technology Corporation, Sterling, VA, United States), cutting through six sections of cooked meat.

The fixed breast muscle samples were embedded in paraffin blocks. Sections (5 μm) were stained with hematoxylin and eosin (H&E). Muscle fiber density and diameter were measured using SlideProcess software, as described previously (16).

### 2.6 Determination of biochemical parameters in plasma

Alanine aminotransferase (ALT), albumin (ALB), aspartate aminotransferase (AST), calcium (Ca), creatinine (CREA), free fatty acids (FFA), glucose (GLU), high-density lipoprotein-cholesterol (HDL-C), low-density lipoprotein-cholesterol (LDL-C), total bile acids (TBA), total cholesterol (TC), total protein (TP), triglycerides (TG), and uric acid (UA) were measured using commercial kits and an automatic biochemical analyzer (Beckman Company, Brea, CA, United States), following the manufacturer’s protocols.

### 2.7 Determination of antioxidant capacity in plasma and muscle

Plasma and muscle samples were preprocessed according to the standardized protocols provided with the commercial assay kits. Antioxidant capacity indicators, including malondialdehyde (MDA), glutathione (GSH), glutathione peroxidase (GSH-PX), superoxide dismutase (SOD), and total antioxidant capacity (T-AOC) were measured using commercial kits (Nanjing Jiancheng Bioengineering Institute Ltd., Nanjing, China) and a Microplate Reader Infinite M200 PRO (Tecan Trading GmbH, Männedorf, Switzerland).

### 2.8 RNA extraction and analysis of gene expression

The extraction of total RNA from breast muscle was performed using TransZol reagent (TransGen Biotech, Beijing, China). RNA concentration and quality were evaluated with a Nanophotometer N60 (Implen Gmbh, Munich, Germany). The Evo M-MLV RT Kit (Accurate Biology, Changsha, China) was employed to reverse-transcribe RNA into cDNA. Real-time PCR was conducted using the qPCR Kit (Accurate Biology, Changsha, China) on a LightCycler R 480II Real-Time PCR System (Roche, Basel, Switzerland). Reaction system included 10 μL of cDNA, 0.4 μL of forward primer, 0.4 μL of reverse primer, 5 μL of SYBR® Green Premix (Accurate Biology, Changsha, China), and 3.2 μL of ddH_2_O. RT-PCR cycling conditions were as follows: an initial denaturation at 95°C for 10 min, followed by 40 cycles of denaturation at 95°C for 15 s and annealing at 60°C for 30 s, and a final extension at 72°C for 30 s. The relative expression of target genes was calculated using the 2^-ΔΔCT^ method with *β-actin* as the endogenous reference (17). The sequences of specific primers are provided in Supplementary Table 1.

### 2.9 Statistical analysis

Data analysis was performed with SPSS 22.0 software (IBM Corp., Armonk, NY, United States). The Shapiro–Wilk test was applied to evaluate normality, and Levene’s test was used to assess the homogeneity of variances. When conditions were met, a one-way ANOVA with Tukey’s post-hoc test was conducted to determine significant differences. When conditions were not met, Welch’s ANOVA and Games–Howell tests were performed for significant difference analysis. Data are presented as means ± SEM. Differences were regarded as significant when *p* < 0.05, with trends defined as 0.05 ≤ *p* < 0.10.

## 3 Results

### 3.1 Impact of dietary FSP on carcass characteristics of aged laying hens

Table 3 presented the effects of dietary FSP supplementation on the carcass characteristics of aged laying hens. Compared with the control group, the addition of 0.25% FSP to the diet enhanced (*p* < 0.05) the dressing percentage of laying hens. Additionally, dietary 1% FSP supplementation showed a downward tendency (*p* = 0.081) in the percentage of leg muscle compared with the other groups. No statistically significant differences (*p* > 0.10) were found in other indicators of carcass characteristics in aged laying hens.

**Table 3:** Effects of dietary fermented strawberry pomace (FSP) supplementation on carcass characteristics of aged laying hens.

Item	Control	Dietary FSP level (%)			SEM	*p*-values
		0.25	0.5	1.0		
Live weight (kg)	1.72	1.71	1.71	1.89	0.037	0.248
Slaughter weight (kg)	1.59	1.60	1.59	1.77	0.035	0.216
Eviscerated weight (kg)	1.12	1.09	1.11	1.24	0.028	0.211
Dressing percentage (%)	92.24^b^	93.65^a^	93.04^ab^	93.45^ab^	0.196	0.040
Percentage of eviscerated yield (%)	64.57	63.60	64.78	65.85	0.548	0.599
Percentage of breast muscle (%)	11.96	11.77	11.07	11.46	0.159	0.176
Percentage of leg muscle (%)	13.39	13.22	12.64	11.72	0.252	0.081
Index of liver (g/kg)	17.38	16.31	16.30	17.78	0.521	0.693
Index of spleen (g/kg)	1.08	1.15	1.10	0.91	0.037	0.133
Index of abdominal fat (g/kg)	42.89	48.70	42.65	54.22	2.697	0.424

Data are expressed as means with their SEM (*n* = 8). ^a,b^Mean values without a common superscript letter within the same row are differed significantly at *p* < 0.05.

### 3.2 Impact of dietary FSP on muscle quality of aged laying hens

Table 4 presented the effects of dietary FSP supplementation on the breast and leg muscle quality of aged laying hens. The breast muscle in all treated groups demonstrated lower pH at 48 h and reduced drip loss at both 24 and 48 h compared to the control group (*p* < 0.05). Furthermore, the pH at 48 h was higher (*p* < 0.05) in the breast muscle of the 0.25% FSP group compared with the 1% FSP group. The 0.5% FSP group exhibited enhanced cooking loss in the breast muscle (*p* < 0.05) compared to the control group. In the leg muscle, the 0.25% FSP group had a higher L* value (*p* < 0.05) than the 0.5% FSP group; the a* value was lower (*p* < 0.05) in the 0.25 and 1% FSP groups than in the control group; the 0.5% FSP group exhibited a higher pH at 45 min (*p* < 0.05) compared to the control and 0.25% FSP groups. At 48 h, the pH was higher (*p* < 0.05) in the 0.25 and 0.5% FSP groups in comparison with the control and 1% FSP groups. Dietary supplementation with 0.25% FSP decreased (*p* < 0.05) drip loss in the leg muscle at 48 h in comparison with the control and 1% FSP groups, while supplementing 0.25 and 1% FSP showed a decreasing trend (*p* = 0.061) in drip loss at 24 h compared with the control group. Furthermore, cooking loss in all treated groups was lower (*p* < 0.05) in comparison with the control group.

**Table 4:** Effects of dietary fermented strawberry pomace (FSP) supplementation on meat quality of aged laying hens.

Item	Control	Dietary FSP level (%)			SEM	*p*-values
		0.25	0.5	1.0		
Breast muscle						
a* (redness)	11.27	12.15	10.96	11.40	0.190	0.151
b* (yellowness)	12.49	12.01	11.80	13.04	0.229	0.232
L* (lightness)	53.40	52.01	52.81	52.47	0.298	0.423
pH_45 min_	6.12	6.45	6.27	6.10	0.071	0.284
pH_48 h_	5.94^a^	5.71^b^	5.64^bc^	5.52^c^	0.033	<0.001
24 h Drip loss (%)	6.75^a^	1.79^b^	2.91^b^	3.16^b^	0.420	<0.001
48 h Drip loss (%)	8.43^a^	3.88^b^	4.04^b^	4.35^b^	0.435	<0.001
Cooking loss (%)	14.58^b^	16.83^ab^	18.47^a^	15.92^ab^	0.440	0.009
Shear force	26.12	23.86	27.88	27.46	0.764	0.239
Leg muscle						
a* (redness)	19.37^a^	16.08^b^	17.72^ab^	17.58^b^	0.303	<0.001
b*(yellowness)	7.86	7.74	7.70	8.38	0.202	0.630
L* (lightness)	40.61^ab^	43.61^a^	40.08^b^	41.29^ab^	0.462	0.027
pH_45 min_	6.63^b^	6.71^b^	6.95^a^	6.72^ab^	0.036	0.007
pH_48 h_	5.93^b^	6.20^a^	6.33^a^	6.04^b^	0.019	<0.001
24 h Drip loss (%)	4.46	2.63	3.52	4.04	0.251	0.061
48 h Drip loss (%)	6.25^a^	4.47^b^	5.04^ab^	6.46^a^	0.292	0.038
Cooking loss (%)	32.57^a^	28.33^b^	27.07^b^	28.14^b^	0.482	<0.001

Data are expressed as means with their SEM (*n* = 8). ^a–c^Mean values without a common superscript letter within the same row are differed significantly at *p* < 0.05.

### 3.3 Impact of dietary FSP on muscle fiber characteristics of aged laying hens

**Figure 1:**
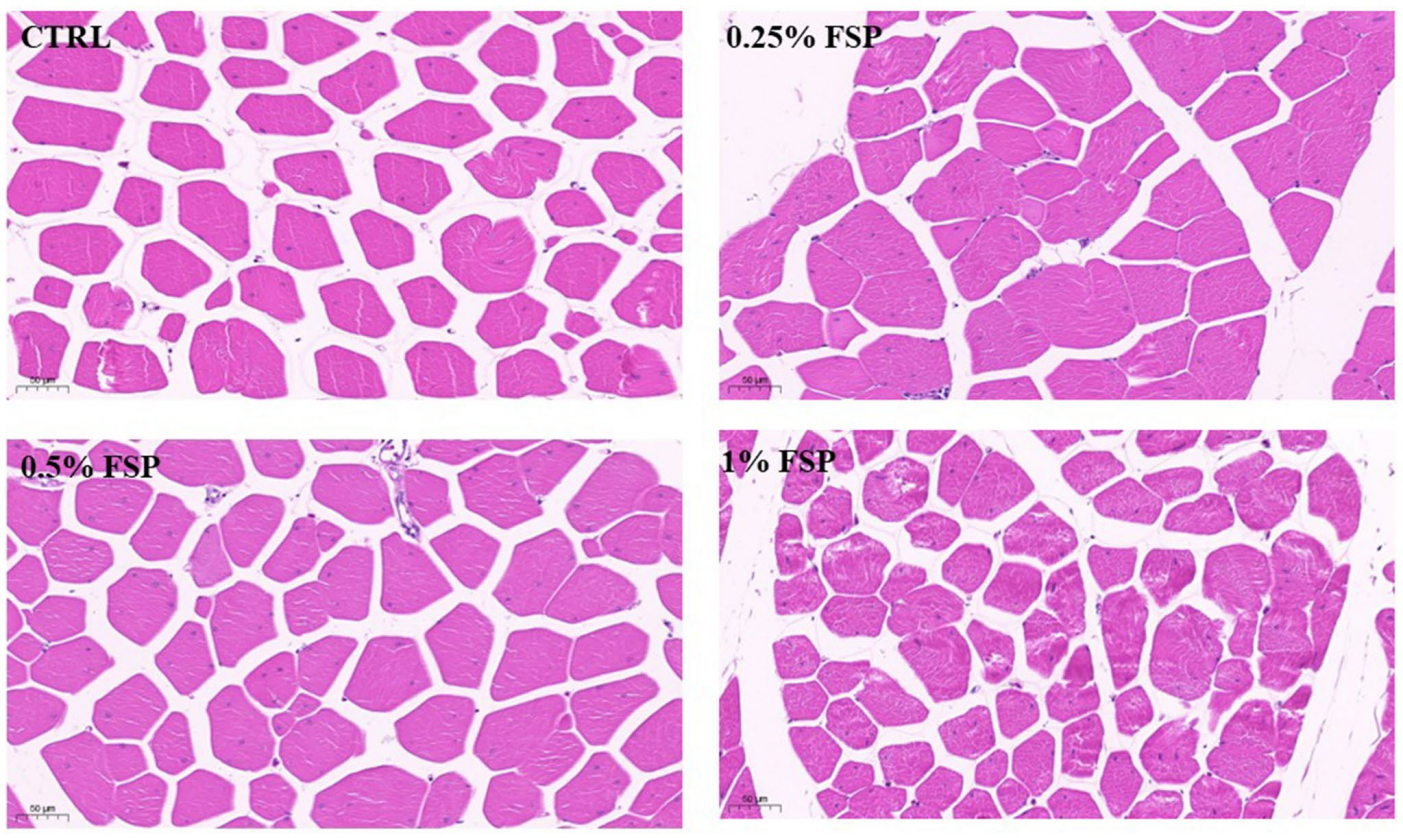
Representative images of H&E stained breast muscle sections illustrate specifics of morphology of the breast muscle fiber of aged laying hen. CTRL, control group; FSP, Fermanted strawberry pomace group; ×400, scale bar 50 μm.

Table 5 presented the impacts of dietary FSP supplementation on muscle fiber characteristics of aged laying hens. FSP exhibited no significant effects on muscle fiber diameter, and no statistical differences in fiber density were observed among groups (*p* > 0.10). Furthermore, morphological examination (Figure 1) revealed no apparent alterations in breast muscle fiber morphology among treatment groups, with fibers maintaining a closely packed arrangement and normal structural integrity.

**Table 5:** Effects of dietary fermented strawberry pomace (FSP) supplementation on density and diameter of the breast muscle fiber of aged laying hens.

Item	Control	Dietary FSP level (%)			SEM	*p*-values
		0.25	0.5	1.0		
Diameter (μm)	71.50	74.05	67.93	70.86	2.160	0.831
Density (number/mm^2^)	157.22	145.33	136.63	144.42	5.222	0.621

Data are expressed as means with their SEM (*n* = 4).

### 3.4 Impact of dietary FSP on plasma biochemical parameters of aged laying hens

Table 6 presented the impacts of dietary FSP supplementation on plasma biochemical parameters of aged laying hens. In comparison with the other three groups, the 1% FSP group had a higher plasma GLU level (*p* < 0.05). The plasma FFA level was reduced (*p* < 0.05) by dietary supplementation with FSP (0.25–1%) compared to the control group. Additionally, supplementing 0.25–1% FSP exhibited a decreasing trend in plasma TG level (*p* = 0.057) in comparison with the control group. No statistically significant differences (*p* > 0.10) were found in the other plasma biochemical parameters.

**Table 6:** Effects of dietary fermented strawberry pomace (FSP) supplementation on plasma biochemical parameters of aged laying hens.

Item	Control	Dietary FSP level (%)			SEM	*p*-values
		0.25	0.5	1.0		
ALB (g/L)	21.13	19.89	18.85	17.36	0.589	0.135
ALT (U/L)	146.44	159.97	150.66	158.00	3.166	0.626
AST (U/L)	170.88	162.14	165.29	152.71	0.444	0.226
Ca (mmol/L)	6.52	6.89	6.37	5.59	0.474	0.114
CREA (μmol/L)	10.56	11.38	11.63	11.88	0.216	0.792
FFA (mmol/L)	0.30^a^	0.15^b^	0.20^b^	0.19^b^	0.013	<0.001
GLU (mmol/L)	14.53^b^	14.06^b^	14.15^b^	15.98^a^	0.177	<0.001
HDL-C (mmol/L)	0.11	0.15	0.38	0.34	0.071	0.534
LDL-C (mmol/L)	0.35	0.25	0.38	0.29	0.022	0.127
TBA (μmol/L)	49.10	38.90	48.21	45.75	3.684	0.776
TC (mmol/L)	4.07	4.00	3.69	3.32	0.206	0.589
TG (mmol/L)	12.83	12.57	12.34	9.89	0.439	0.057
TP (g/L)	58.51	59.41	57.51	54.10	0.474	0.420
UA (mg/dl)	5.12	4.30	5.48	5.29	0.197	0.238

Data are expressed as means with their SEM (*n* = 8). ^a,b^Mean values without a common superscript letter within the same row are differed significantly at *p* < 0.05. ALB, albumin; ALT, alanine aminotransferase; AST, aspartate aminotransferase; Ca, calcium; CREA, creatinine; FFA, free fatty acids; GLU, glucose; HDL-C, high-density lipoprotein-cholesterol; LDL-C, low-density lipoprotein-cholesterol; TBA, total bile acids; TC, total cholesterol; TG, triglycerides; TP, total protein; UA, uric acid.

### 3.5 Impact of dietary FSP on plasma and breast muscle antioxidant capacity of aged laying hens

Table 7 presented the impacts of dietary FSP supplementation on antioxidant capacity of plasma and breast muscle of aged laying hens. Compared with the control and 0.25% FSP groups, the plasma T-AOC level was increased (*p* < 0.05) in the 1% FSP group, while the plasma GSH-PX level was lower (*p* < 0.05) in the 0.5 and 1% FSP groups. Moreover, the MDA level in the breast muscle of the 0.25% FSP group was lower (*p* < 0.05) than that of the 1% FSP group.

**Table 7:** Effects of fermented strawberry pomace (FSP) on the antioxidant capacity of plasma and breast muscle of aged laying hens.

Item	Control	Dietary FSP level (%)			SEM	*p*-values
		0.25	0.5	1.0		
Plasma						
GSH (U/mg prot)	0.61	0.99	0.32	0.53	0.093	0.069
GSH-PX (U/mg prot)	1167.38^a^	1056.92^a^	792.43^b^	721.96^b^	42.971	<0.001
MDA (nmol/mg prot)	9.33	10.34	11.08	11.13	0.414	0.506
SOD (U/mL)	327.48	382.62	395.24	368.83	14.017	0.360
T-AOC (mM/mg)	0.56^b^	0.68^b^	0.75^ab^	1.13^a^	0.061	0.003
Breast muscle						
GSH (U/mg prot)	0.80	1.31	1.48	0.93	0.129	0.212
GSH-PX (U/mg prot)	8.57	4.77	10.91	11.21	0.531	0.422
MDA (nmol/mg prot)	0.27^ab^	0.17^b^	0.30^ab^	0.47^a^	0.033	0.010
SOD (U/mL)	7.86	7.24	7.84	7.20	0.256	0.697
T-AOC (mM/mg)	0.07	0.07	0.07	0.05	0.003	0.243

Data are expressed as means with their SEM (*n =* 8). ^a,b^Mean values without a common superscript letter within the same row are differed significantly at *p* < 0.05. GSH, glutathion; GSH-PX, glutathione peroxidase; MDA, malondialdehyde; SOD, superoxide dismutase; T-AOC, total antioxidant capacity.

### 3.6 Impact of dietary FSP on gene expressions related to antioxidant capacity in breast muscle of aged laying hens

Table 8 presented the impacts of dietary FSP supplementation on the expression of antioxidant-related genes in the breast muscle of aged laying hens. In comparison with the control and 0.25% FSP groups, the expression of *SOD1* was up-regulated (*p* < 0.05) in the 0.5% FSP group. All treated groups up-regulated the expression of myosin heavy chain *MYH1B* (*p* < 0.05), and the expression of *MYH1G* showed an upregulating trend (*p* = 0.073) in the 0.25 and 0.5% FSP groups compared with the control group.

**Table 8:** Effects of dietary fermented strawberry pomace (FSP) supplementation on the breast muscle antioxidant gene expression of aged laying hens.

Item	Control	Dietary FSP level (%)			SEM	*p*-values
		0.25	0.5	1.0		
*CAT*	1.00	0.96	1.27	1.15	0.056	0.200
*GPX1*	1.00	1.11	1.11	1.01	0.050	0.793
*HO-1*	1.00	1.06	1.03	1.17	0.056	0.724
*Keap1*	1.00	0.92	1.07	1.01	0.044	0.676
*MYF5*	1.00	0.96	1.09	0.94	0.040	0.550
*MYH1B*	1.00^b^	1.77^a^	1.68^a^	1.70^a^	0.071	< 0.001
*MYH1G*	1.00	1.32	1.27	1.13	0.048	0.073
*NQO1*	1.00	1.03	1.13	1.00	0.050	0.795
*Nrf2*	1.00	0.95	1.12	0.95	0.043	0.440
*SOD1*	1.00^b^	1.11^b^	1.60^a^	1.42^ab^	0.073	0.007
*SOD2*	1.00	1.20	1.23	1.13	0.050	0.397

Data are expressed as means with their SEM (*n* = 8). ^a,b^Mean values without a common superscript letter within the same row are differed significantly at *p* < 0.05. *CAT*, catalase; *GPX1*, glutathione peroxidase 1; *HO-1*, heme oxygenase-1; *Keap1*, kelch-like ECH-associated protein 1; *MYF5*, myogenic factor 5; *MYH1B*, myosin, heavy chain 1B, skeletal muscle; *MYH1G*, myosin, heavy chain 1G, skeletal muscle; *NQO1*, NAD(P)H quinone oxidoreductase 1; *Nrf2*, nuclear factor erythroid 2-related factor 2; *SOD1*, superoxide dismutase 1; *SOD2*, superoxide dismutase 2.

## 4 Discussion

The findings showed that FSP supplementation as a feed additive enhanced carcass characteristics, improved meat quality, and increased the antioxidant capacity of aged laying hens.

Carcass characteristics is an important metric for assessing the economic benefits of livestock production. In this study, 0.25% FSP supplementation increased the dressing percentage of aged laying hens compared to the control group. However, no statistically significant differences in dressing percentage were found among the different FSP supplementation levels, suggesting that a lower dose was sufficient to improve the dressing percentage. A previous study reported that 3% dried strawberry pomace improved the dressing percentage of broiler chickens (17). However, another study indicated that dietary treatments with 3 and 6% dried strawberry pomace did not alter dressing percentage (18). This difference might be explained by the fact that fermentation enhances the nutritional value of strawberry pomace. A decreasing trend in leg muscle percentage was observed as the FSP dose decreased, suggesting that high-dose FSP may not be beneficial for increasing leg muscle yield.

Meat quality is critical because it affects shelf life, taste, flavor, and consumer preferences (19). Previous research has demonstrated that lipid and protein oxidation reduce water-holding capacity (WHC) in meat by altering membrane structures and compositions, and by affecting protein fragmentation and aggregation (20, 21). Drip loss, as an indicator of meat WHC, plays a vital role in both the meat industry and consumer evaluation of meat quality. In this study, dietary FSP supplementation reduced the 24-h and 48-h drip loss of the breast muscle; the 24-h drip loss of leg muscle tended to decrease in the 0.25–1% FSP groups, and the 48-h drip loss decreased in the 0.25% FSP group compared to the control and 1% FSP groups. These results suggest that dietary FSP improves WHC in both breast and leg muscles. These findings align with a previous study (17), which reported that strawberry pomace reduces drip loss in breast and leg muscles. Antioxidants serve as protective agents by neutralizing reactive oxygen species, thereby safeguarding organelles, cells, and tissues from oxidative damage (22, 23). Therefore, the decline in drip loss can be due to the high content of diverse antioxidants present in strawberry pomace, including vitamins, polyphenols, and anthocyanins, which protect against oxidative cell damage.

After slaughter, lactic acid production in muscle from glycolysis causes pH reduction, and this pH decrease is positively linked to meat quality traits like tenderness, WHC, color, juiciness, and shelf life. A higher pH indicates improved WHC, reduced cooking loss, and darker muscle color (24). Several previous studies have found that strawberry pomace supplementation did not affect pH values (18, 25). However, in our study, dietary FSP (0.25–1%) supplementation decreased the pH at 48 h in the breast muscle. In addition, the pH at 45 min in the leg muscle was increased in the 0.5% FSP group compared with the control and 0.25% FSP groups, while the pH at 48 h in the leg muscle was higher in the 0.25 and 0.5% FSP groups compared with the control and 1% FSP groups, indicating that dietary FSP can improve leg meat quality. Furthermore, 0.5% FSP supplementation increased the breast muscle cooking loss compared with the control group, which might be attributed to the decreased pH in the breast muscle. Moreover, the leg muscle cooking loss was significantly reduced with 0.25–1% FSP supplementation compared to the control group, possibly due to the increased pH, suggesting a beneficial effect on leg muscle quality.

To further understand the changes in meat quality, the myofiber profiles in the breast muscle were examined. However, there were no significant differences between the control and all treated groups. In the process of postmortem glycolysis, skeletal muscle converts stored glycogen into ATP, lactate, and ultimately hydrogen ions. The formation of hydrogen ions results in a drop in muscle pH (26). FSP may have altered the glycogen content in different skeletal muscles by influencing plasma glucose level, thereby affecting muscle pH. However, the specific mechanisms require further investigation.

Meat color reflects freshness and quality (27). Strawberry pomace is rich in natural bioactive components, such as carotenoids and anthocyanins, which may influence meat color (28). In the present study, 0.5% FSP supplementation increased the L* value in the leg muscle compared to the 0.25% FSP group. Moreover, dietary 0.25 and 1% FSP decreased the a* value in the leg muscle compared to the control group. However, Colombino et al. (18) indicated that the addition of 3 and 6% strawberry pomace to broilers’ diets did not affect meat color. Similarly, Juśkiewicz et al. (25) reported that supplementing 5% dried strawberry pomace in the diet had no effect on the color parameters of turkeys. However, Sosnówka-Czajka et al. (17) found that 3% strawberry pomace supplementation tended to decrease the a* value in the breast muscle of broiler chickens. These findings suggest that a higher dose of strawberry pomace may decrease the color quality of leg muscle. The discrepancy between these findings and our results may result from differences in chicken breeds and the doses used. Further investigation is warranted to elucidate the specific reasons.

Plasma biochemical parameters directly indicate animal health and metabolic status. Nordestgaard and Varbo (29) indicated that increased TG content is associated with several diseases, such as cardiovascular disease, acute pancreatitis, and atherosclerosis. In the present study, 1% FSP supplementation showed a decreasing trend in plasma TG content, suggesting that dietary FSP might improve lipid metabolism and benefit overall health. Additionally, 1% FSP supplementation was found to be most effective for increasing GLU content, which may imply that a higher dose of FSP could aid in carbohydrate digestion and absorption (30). Plasma FFA level is elevated in conditions such as obesity, non-alcoholic fatty liver disease, insulin resistance, cardiovascular disease, and type 2 diabetes (31). In the present study, plasma FFA levels decreased in all treated groups, indicating positive effects on metabolic status. However, several previous studies have reported no impacts of tomato pomace, grape pomace, or papaya pomace on plasma GLU, FFA, or TG levels (32–34). These findings suggest that strawberry pomace exhibits greater antioxidant activity than other fruit pomaces, likely due to its unique nutritional composition (35).

Oxidative rancidity is a common cause of quality deterioration in food, primarily due to the oxidative deterioration of lipids. This process not only produces unpleasant odors but also leads to changes in flavor, texture, consistency, appearance, and nutritional value in meat (36). Antioxidant enzymes are crucial for maintaining the redox balance in the body and supporting normal organ function. Increasing the activity of these enzymes is an effective strategy to reduce oxidative stress and delay oxidation processes, thereby maintaining optimal quality and extending shelf life (37). Strawberry pomace has demonstrated beneficial antioxidant activity, enhancing the nutritional value and health advantages of animal products for consumers (38, 39). In the present study, plasma T-AOC level increased in the 1% FSP group, consistent with the findings of the Qin et al. (12). These results suggest that antioxidants in FSP, such as vitamin C and anthocyanins, may modulate the enzymatic system in plasma by increasing T-AOC level. However, plasma GSH-PX level decreased in the 0.5 and 1% FSP groups, possibly due to increased oxidative stress in certain tissues, which led to excessive consumption or damage of GSH-PX in those regions. Despite this, the overall antioxidant capacity increased due to enhanced antioxidant status in other areas. MDA is a widely accepted biomarker of oxidative stress, particularly lipid peroxidation (40). In the present study, MDA level in breast muscle was significantly reduced in the 0.25% FSP group compared to the 1% FSP group, suggesting that low-dose FSP may alleviate lipid peroxidation and improve meat quality.

SOD is commonly recognized as an enzyme that scavenges free radicals, playing a critical role in antioxidant defense through driving the conversion of superoxide anions to hydrogen peroxide during cellular antioxidant processes (41). The Cu and Zn-containing enzymes (Cu-SOD and Zn-SOD), known as SOD1, is located in the cytosol, intermembrane space of mitochondria, and nucleus (42). In the present study, 0.5% FSP supplementation significantly up-regulated *SOD1* expression in the breast muscle compared to the control and 0.25% FSP groups, indicating an enhancement in the breast muscle’s antioxidant capacity.

Increasing the proportion of slow fibers in muscle can enhance the color and water retention capacity of fresh meat. *MYH1B* interacts with lncRNA-FKBP1C to stabilize muscle protein, promoting the conversion from fast to slow muscle fibers (43). In the present study, FSP supplementation up-regulated *MYH1B* expression in the breast muscle, indicating a reduction in drip loss, which is consistent with earlier findings in this study.

## 5 Conclusion

Dietary FSP supplementation improves carcass characteristics, meat quality, plasma lipid profiles, and plasma antioxidant capacity in aged laying hens by enhancing muscle antioxidant capacity and muscle fiber type. Notably, low-dose FSP synergistically enhanced production efficiency and meat quality, with 0.25% improving dressing percentage, reducing oxidative damage (lower MDA), and minimizing drip loss, while 0.5% upregulating antioxidant (*SOD1*) and myogenic (*MYH1B*) genes, suggesting optimal bioactive compound utilization. In contrast, 1% FSP paradoxically compromised muscle yield (leg muscle decline) and disrupted redox balance (elevated T-AOC but suppressed GSH-PX), indicating potential metabolic overload. Further research is needed to determine the optimal dosage of FSP for maximizing benefits while minimizing adverse effects. These findings offer a valuable reference for the cost-effective use of dietary FSP in poultry rearing and the utilization of fruit-processing by-products.

## Data Availability

The original contributions presented in the study are included in the article/Supplementary material. Further inquiries can be directed to the corresponding author.
