# Supplementary material for: Fermented strawberry pomace enhances carcass characteristics and meat quality by regulating plasma biochemical indices and antioxidant capacity in aged laying hens
Source: Front Nutr. 2025 Jun 19;12:1610660. doi: 10.3389/fnut.2025.1610660 (PMC12221941; doi:10.3389/fnut.2025.1610660)
Supplement: Supplementary file 1 [file Table_1.DOCX]

**Supplemental Table 1.** Primer sequences of genes for RT-qPCR.

| **Gene** | **Accession number** | **Primer sequence (5′−3′)** | **Product size (bp)** |
| --- | --- | --- | --- |
| *CAT* | NM_001031215.2 | F: AGATGGCGTATGACCCTAGC  R: CCTCTGATAATTGGCCACGC | 173 |
| *GPX1* | NM_001277853.2 | F: ATGTTCGAGAAGTGCGAGGT  R: AGTTCCAGGAGACGTCGTTG | 160 |
| *HO–1* | NM_205344.1 | F: ATGCCTACACCCGCTATTTG  R: ATCTCAAGGGCATTCATTCG | 178 |
| *Keap1* | MN416132.1 | F: CATCAACTGGGTGCAGTACG  R: AGGGTGAGGTCCTGGAAGAT | 183 |
| *MYF5* | NM_001030363.2 | F: CAGCCACTATGAGGGAGAGG  R: ACCTGTTCCCTCAAGAGCTC | 166 |
| *MYH1B* | NM_204228.4 | F: GCGGCTGAAGAAGAAGATGG  R: TCCTCAATCTCAGCCTGCAA | 225 |
| *MYH1G* | NM_001395983.1 | F: AGAATCGAGGCCCAGAACAA  R: ACTTGGGAGGGTTCATGGAG | 184 |
| *NQO1* | NM_001277619.1 | F: AAGAAGATTGAAGCGGCTGA  R: GCATGGCTTTCTTCTTCTGG | 166 |
| *Nrf2* | NM_205117.1 | F: CCACCCTAAAGCTCCATTCA  R: ATTCTTGCCTCTCCTGCGTA | 217 |
| *SOD1* | NM_205064.1 | F: ATTACCGGCTTGTCTGATGG  R: CCTCCCTTTGCAGTCACATT | 173 |
| *SOD2* | NM_204211.2 | F: CCTTCGCAAACTTCAAGGAG  R: CCAGCAATGGAATGAGACCT | 162 |
| *β–actin* | NM_205518.1 | F: ATGAAGCCCAGAGCAAAAGA  R: GGGGTGTTGAAGGTCTCAAA | 223 |

Note: *CAT, catalase; GPX1, glutathione peroxidase 1; HO-1, heme oxygenase-1; Keap1, kelch-like ECH-associated protein 1; MYF5, myogenic factor 5; MYH1BMyHCIb, myosin, heavy chain 1B, skeletal musclemyosin, heavy chain 1, skeletal muscle, bounding; MYH1G, myosin, heavy chain 1G, skeletal musclemyosin, heavy chain 1, skeletal muscle, gene; NQO1, NAD(P)H quinone oxidoreductase 1NAD(P)H: quinone oxidoreductase; Nrf2, nuclear factor erythroid 2-related factor 2; SOD1, superoxide dismutase 1; SOD2, superoxide dismutase 2.*
